# Old Yellow Enzyme homologues in *Mucor circinelloides*: expression profile and biotransformation

**DOI:** 10.1038/s41598-017-12545-7

**Published:** 2017-09-21

**Authors:** Alice Romagnolo, Federica Spina, Anna Poli, Sara Risso, Bianca Serito, Michele Crotti, Daniela Monti, Elisabetta Brenna, Luisa Lanfranco, Giovanna Cristina Varese

**Affiliations:** 1 Department of Life Sciences and Systems Biology, University of Turin, viale P. A. Mattioli 25, 10125 Turin, Italy; 2 Department of Chemistry, Materials and Chemical Engineering “G. Natta”, Politecnico di Milano, via L. Mancinelli 7, 20131 Milan, Italy; 3 Istituto di Chimica del Riconoscimento Molecolare, CNR, Via M. Bianco 9, 20131 Milan, Italy

## Abstract

The reduction of C=C double bond, a key reaction in organic synthesis, is mostly achieved by traditional chemical methods. Therefore, the search for enzymes capable of performing this reaction is rapidly increasing. Old Yellow Enzymes (OYEs) are flavin-dependent oxidoreductases, initially isolated from *Saccharomyces pastorianus*. In this study, the presence and activation of putative OYE enzymes was investigated in the filamentous fungus *Mucor circinelloides*, which was previously found to mediate C=C reduction. Following an *in silico* approach, using *S. pastorianus* OYE1 amminoacidic sequence as template, ten putative genes were identified in the genome of *M. circinelloides*. A phylogenetic analysis revealed a high homology of McOYE1-9 with OYE1-like proteins while McOYE10 showed similarity with thermophilic-like OYEs. The activation of *mcoyes* was evaluated during the transformation of three different model substrates. Cyclohexenone, α-methylcinnamaldehyde and methyl cinnamate were completely reduced in few hours and the induction of gene expression, assessed by qRT-PCR, was generally fast, suggesting a substrate-dependent activation. Eight genes were activated in the tested conditions suggesting that they may encode for active OYEs. Their expression over time correlated with C=C double bond reduction.

## Introduction

The reduction of C=C double bonds is a key reaction in organic chemistry but it is usually carried out by metal catalysts with a strong impact on the technical and economic feasibility of the process^1,2^. For instance, Yang *et al*.^3^ reported that toxic traces of heavy metals remained in the reaction products and needed to be removed before pharmaceutical use.

Since the major challenges of bulk and fine chemicals synthesis are the reduction of the environmental impact and process costs, biocatalysis became one of the most intriguing alternative to traditional processes. The use of microorganisms or their enzymes has recently found room in the industrial production of pharmaceuticals, flavors, aromas, etc.^2,4^. The biological reduction of activated C=C double bonds may be carried out by flavin-dependent oxidoreductases, namely ene reductases (ERs), belonging to the Old Yellow Enzyme (OYE) family (EC 1.6.99.1)^5^. They catalyze the asymmetric hydrogenation of C=C double bond conjugated with electron withdrawing groups (EWGs) in the presence of NAD(P)H as cofactor^2^. In contrast with heavy metals, which are capable of mediating *cis*-hydrogenation, OYEs can catalyze this reaction *trans*-fashion with high stereo-selectivity^1^.

The reactions catalyzed by OYEs are very interesting and have strong application outcomes. Robinson and Panaccione^6^ showed the involvement of OYE homologues involvement in the biosynthetic pathway of ergot alkaloids, commonly used to treat disorders such as Alzheimer’s disease, dementia, type 2 diabetes, and hyperprolactinemia or to induce labor and reduce bleeding (lysergic acid-derived drugs). OYE1 from *S. pastorianus* transformed methyl 2-hydroxymethylacrylate in (*R*)-3-hydroxy-2-methylpropanoate, known as “Roche-Ester”, which is a chiral building block for the synthesis of vitamins (vitamin E)^4^. Some fragrance compounds (muscone), antibiotics (rapamycin), and natural products have been obtained by OYE-mediated reduction^2^. The 12-oxophytodienoate reductase enzymes (OPRs, EC 1.3.1.42), OYE homologues from plants, are involved in the biosynthesis of jasmonic acid, which is implicated in the regulation of plant responses to abiotic and biotic stresses as well as plant growth and development^7^. Pentaerythritol tetranitrate reductase (PETNR) from *Enterobacter cloacae* successfully degraded tri nitro toluene (TNT)^8^.

OYEs have been ubiquitously described in yeasts, bacteria, animals and plants, and recently in filamentous fungi^9^. Fungi are perfect candidates to set up biocatalysis processes: they combine operative versatility to simple growth conditions and they are a well-known enzymatic machinery^1,2,4,10^. For instance, a homologue of OYE has been discovered in *Aspergillus fumigatus* and *Claviceps purpurea* and associated to the ergot biosynthesis^6,11^. To date, most of the literature evidences focused on Ascomycetes and Basidiomycetes^9,12^ but the presence of OYE homologue within Zygomycota phylum has never been assessed.

Despite the potential application in several biotechnological fields, microorganisms and enzymes are still scarcely used in manufacturing processes, mostly due to the lack of suitable biocatalysts. Novel enzymatic activities with strong catalytic potential could be achieved with traditional functional screening or advanced molecular approaches^2,4^. Genome-wide analysis is a useful tool to identify OYEs homologues among the available fungal genomes. For instance, Nizam *et al*.^9^ by analysing 60 Ascomycota and Basidiomycota genomes identified 424 OYEs homologues and provided a first classification of these enzymes within the fungal kingdom. They also explored the evolutionary significance of fungal OYEs. Unfortunately, this data can be considered just a first step, and the actual capability of strains to transform target compounds by reducing C=C double bond need further validation.

In this work, we aimed to fill the lack of information about the occurrence of OYEs in fungi belonging to the Zygomycota phylum. *Mucor circinelloides* was selected due to its ability of converting several substrates^13^. Despite those interesting results, the enzymatic pattern responsible for the reactions has never been investigated before. The availability of *M. circinelloides* complete genome sequence (Joint Genome Institute, JGI: http://jgi.doe.gov) allowed a genome-mining approach to investigate the presence of putative OYEs homologues.

## Results

### Identification of putative OYEs in the genome of *M. circinelloides*

In order to identify OYE encoding genes in the filamentous fungus *M. circinelloides*, a BlastP analysis (Basic Local Alignment Search Tool, NCBI, USA) on the complete genome of *M. circinelloides* using *S. pastorianus* OYE1 as query was performed. Ten putative sequences were retrieved and named McOYE1-McOYE10 (Table 1). The 10 amino acid sequences and the amino acid sequence of OYE1 were aligned to evaluate sequence similarities (Table 1). Nine McOYEs showed a similarity with *S. pastorianus* OYE1 of about 40% while McOYE10 showed a lower similarity (25.33%; Table 1).

**Table 1:** Putative OYE homologues of *M. circinelloides* - McOYE1, McOYE2, McOYE3, McOYE4, McOYE5, McOYE6, McOYE7, McOYE8, McOYE9 and McOYE10 - with sequence ID according to JGI database and identity percentage with *S. pastorianus* OYE1.

McOYE	Sequence ID	ID matrix (%) with OYE1	ID matrix (%) with McOYE1	ID matrix (%) with McOYE2	ID matrix (%) with McOYE3	ID matrix (%) with McOYE4	ID matrix (%) with McOYE5	ID matrix (%) with McOYE6	ID matrix (%) with McOYE7	ID matrix (%) with McOYE8	ID matrix (%) with McOYE9	ID matrix (%) with *McOYE10*
1	160302	44.14	97.50	89.60	70.40	75.00	67.80	66.10	60.60	67.00	58.40	37.00
2	137297	43.99	89.60	97.50	69.70	75.30	68.70	66.10	61.10	67.00	59.50	36.40
3	177510	42.19	71.20	70.50	96.40	76.10	63.30	63.60	57.10	61.60	60.50	40.70
4	155592	41.30	73.40	73.70	73.60	100.00	65.30	65.70	57.80	64.80	60.10	41.60
5	110873	43.41	65.80	66.70	60.40	65.30	100.00	64.50	61.60	65.90	59.10	39.10
6	144573	42.30	64.40	64.30	61.10	64.90	64.60	100.00	67.70	66.10	58.20	38.20
7	153280	41.80	63.70	64.20	62.10	62.20	66.30	68.80	90.50	60.50	56.60	45.10
8	76836	42.19	65.70	65.70	59.50	64.80	65.90	66.10	56.70	100.00	59.20	—
9	134845	38.19	56.90	57.90	57.90	59.50	57.70	57.50	54.90	58.50	97.00	—
10	152500	25.33	35.90	35.20	39.20	41.60	39.10	40.30	48.40	41.60	41.80	100.00

Three conserved domains typical of OYEs were found in all the 10 sequences: the FMN binding site, the active site and the substrate binding site (Supp. Figure 1).

Specific primer pairs were designed on the nucleotide sequences of the 10 putative *mcoyes* (Supp. Table 1) and tested by conventional PCR on genomic DNA. Amplicons of the expected size (about 200 bp) were obtained (Supp. Figure 2). PCR products were sequenced confirming the specificity of the primers pairs and the authenticity of the DNA sequences.

**Figure 1:**
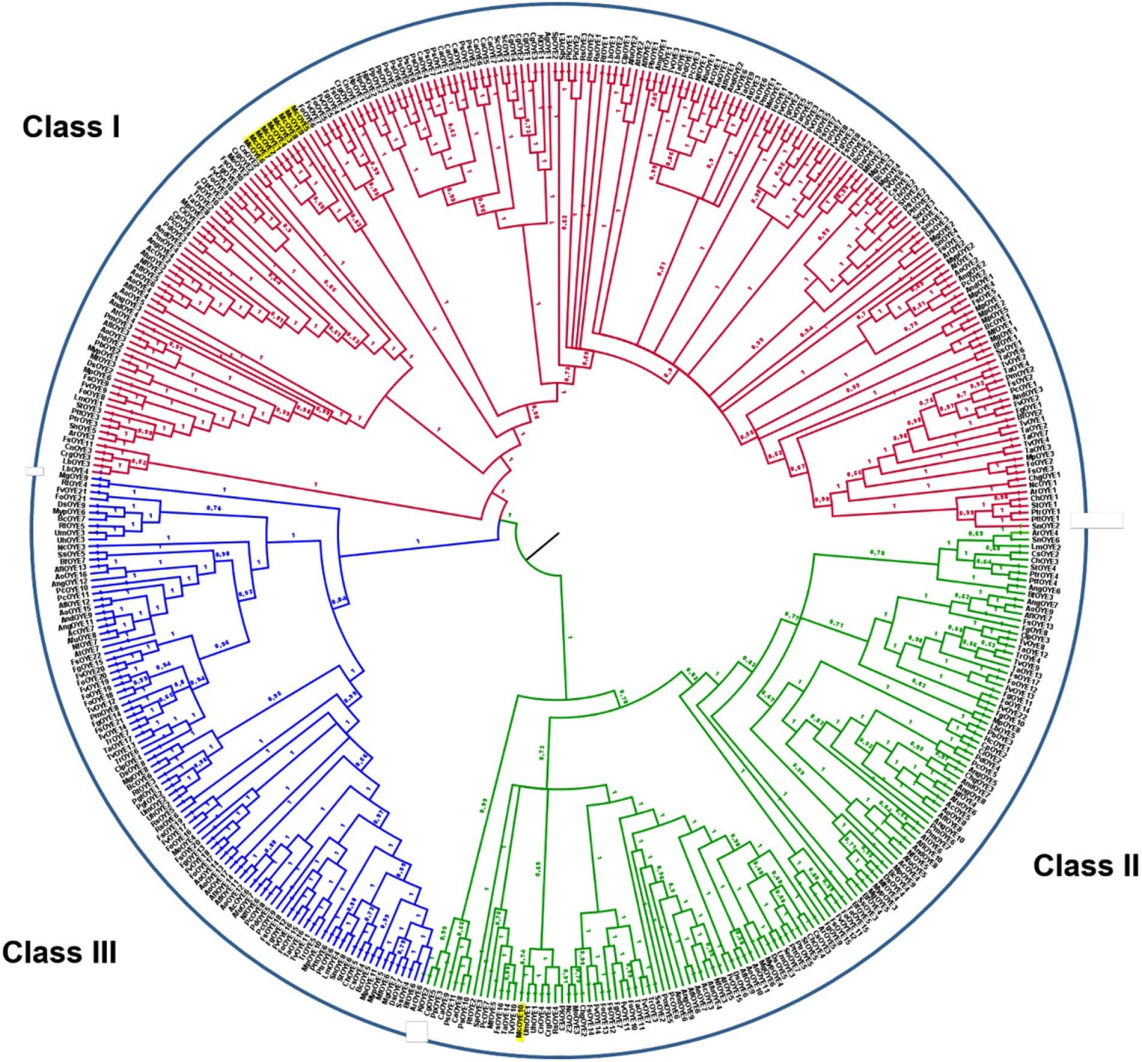
Evolutionary relationship of deduced OYE proteins based on Bayesian inference analysis of the structure-based amino acid sequence alignment. The numbers at the nodes indicates Bayesian posterior probabilities. The phylogenetic tree was implemented from Nizam *et al*.^9^.

A phylogenetic analysis was performed by implementing sequence data analyzed by Nizam *et al*.^9^, who divided OYEs proteins into three groups: Class I, Class II and Class III. Nine out of 10 McOYEs clustered together in Class I showing a specie-specific clade whereas McOYE10 was located within Class II (Fig. 1).

### Biotransformation of conventional substrates and gene expression

The expression profile of the 10 putative OYEs homologues was monitored on the RNA extracted from the mycelium grown in liquid culture during the biotransformation of three conventional substrates presenting different EWGs. For each substrate, data on biotransformation and gene expression pattern are presented. Since the substrate was dissolved in dimethyl sulfoxide (DMSO), *mcoyes* activation was also evaluated in the presence of this solvent to exclude artifacts. None of the genes was activated in the presence of DMSO (data not shown). The 10 genes showed a basal activity in the absence of substrates (data not shown).

#### Cyclohexenone (CE)

**Figure 2:**
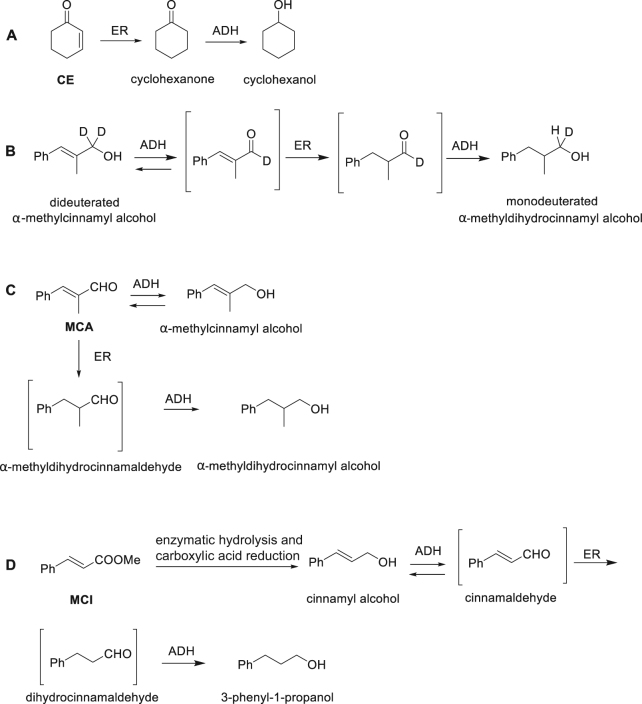
Reaction profiles of (**A**) CE, (**B**,**C**) MCA and (**D**) MCI biotransformations.

**Figure 3:**
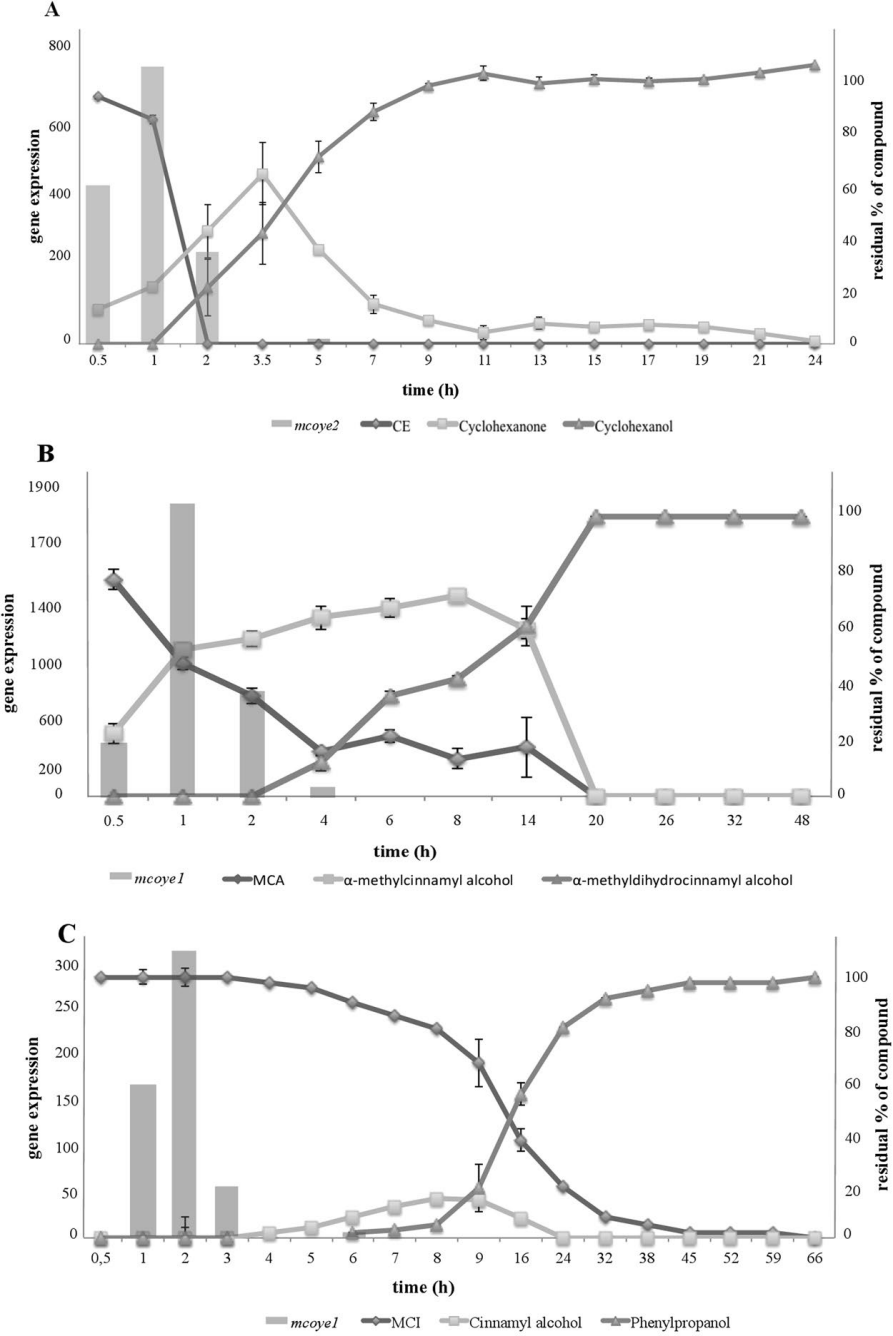
Graph combining the data of expression of (**A**) *mcoye2* in presence of CE (bars) with the biotransformation data of CE (lines); (**B**) *mcoye1* in presence of MCA (bars) with the biotransformation data of MCA (lines); (**C**) *mcoye1* in presence of MCI (bars) with the biotransformation data of MCI (lines). Data are the averages ± standard deviations (error bars) of the results of at least three different biological replicates.

*M. circinelloides* completely reduced the substrate CE into cyclohexanol within 24 h; the reaction process is well known: first an OYE reduces the C=C double bond of CE producing cyclohexanone, then the keto group is reduced by an alcohol dehydrogenase (ADH) into cyclohexanol (Fig. 2A)^13^. As shown in Fig. 3A, the reaction began 30 min after the addition of CE to the medium and at 3.5 h the C=C double bond was completely reduced producing cyclohexanone which was continuously converted in its corresponding alcohol, cyclohexanol.

**Figure 4:**
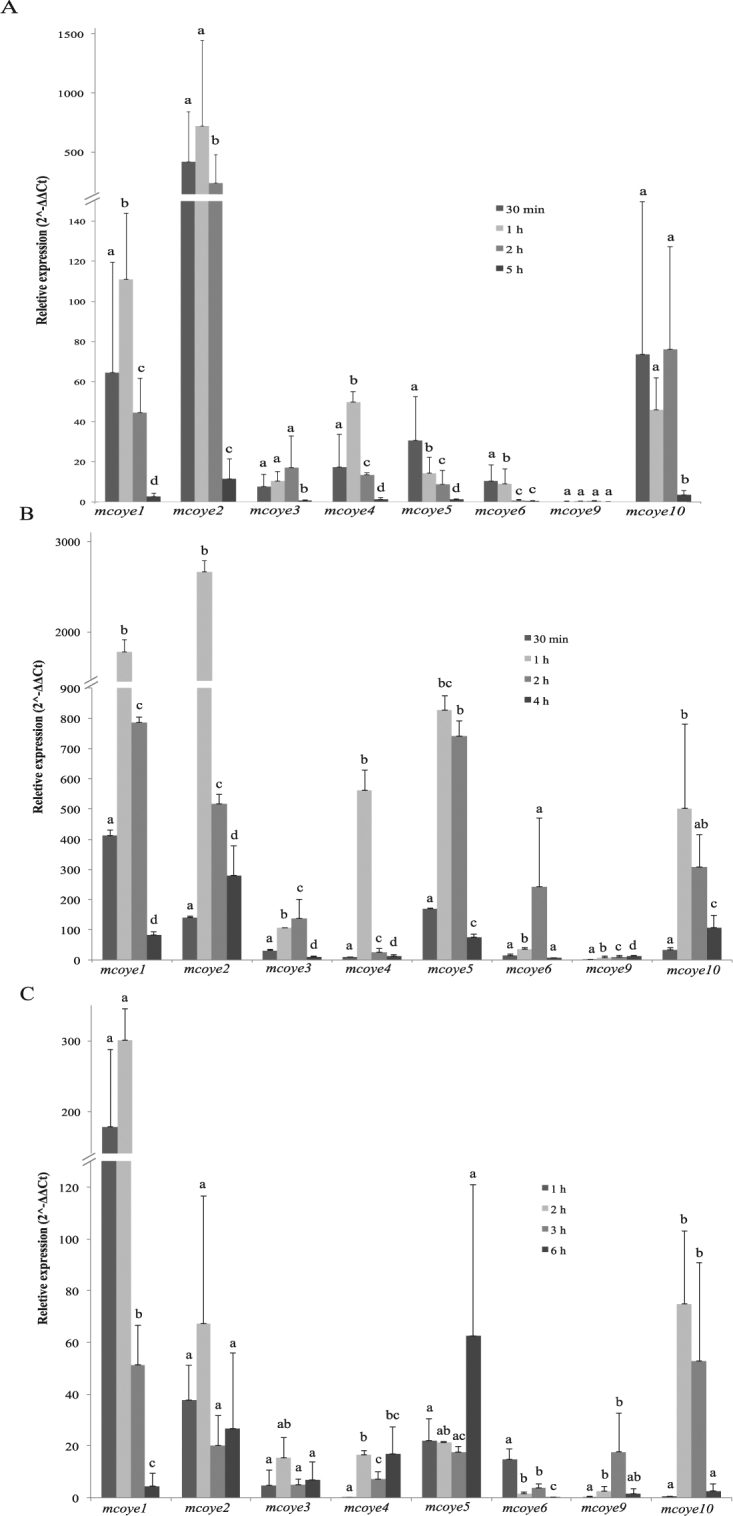
Gene expression of OYE homologues in presence of (A) CE, (B) MCA and (C) MCI during the time course experiments. The relative gene expression was calculated with the 2^−ΔΔCt^ method according to Livak & Schmittgen^27^ using the β-actin as housekeeping gene^26^ and the control (non treated) as reference sample. Different letters indicate statistically significant difference (p < 0.05, ANOVA and Tukey’s tests) for each gene at the different time points.

The transcripts level of the 10 *mcoye* homologues was monitored both in the presence and absence of CE at 30 min, 1 h, 2 h and 5 h (Fig. 4A). With the exception of *mcoye7* and *mcoye8* that did not show activation upon CE exposure (data not shown), all the other genes were activated within the first two hours. In particular *mcoye*2*, mcoye1* and *mcoye10* displayed a fast and strong induction of gene expression: 730, 111 and 76 fold compared to the control sample without CE at 1 h and at 30 min for *mcoye10* (Fig. 4A). *Mcoye4* and *mcoye5* showed an activation of 30–50 fold, while for *mcoye*3, *mcoye6* and *mcoye9* the induction of gene expression compared to the control sample remained below 20 fold at the different time points. Noteworthy, expression levels of these genes decreased to the control values after 5 h.

A clear relation between *mcoyes* expression profile and the biotransformation of CE was observed (Fig. 3A). *Mcoye2* transcript levels were strongly induced at the beginning of the reductive process reaching the maximum at 1 h when 20% of CE had been converted into cyclohexanone.

#### α-Methylcinnamaldehyde (MCA)

*M. circinelloides* completely reduced the C=C double bond of MCA within 20 h. Both the C=C double bond (OYE) and the aldehydic group (ADH) of the substrate were reduced (Fig. 3B). One hour after MCA addition, α-methylcinnamyl alcohol represented 50% of the substrates in the culture medium. The concentration of this first product increased until 8 h and then dropped down before 20 h. The production rate of the saturated alcohol was constant, starting from 2 h until 20 h, when it was the remaining metabolite detected (Fig. 3B).

According to literature data^2^, OYEs are able to catalyze the reduction of the C=C double bonds of unsaturated aldehydes, whereas they are usually inactive on allylic alcohols. In the case of *M. circinelloides*, α-methylcinnamyl alcohol seemed to be the intermediate of the conversion of MCA into the corresponding saturated alcohol. Thus, α-methylcinnamyl alcohol was added directly to *M. circinelloides* cultures, and indeed its conversion into α-methyldihydrocinnamyl alcohol was observed to be complete after 48 h. In order to elucidate this reduction pathway, the dideuterated α-methylcinnamyl alcohol, showing two deuterium atoms linked to the carbon atom bearing the OH group, was prepared (Fig. 2B). This compound was submitted to bioreduction with *M. circinelloides* and a monodeuterated saturated alcohol was recovered. The formation of this compound could be explained only admitting the formation of the unsaturated aldehyde as an intermediate, because the two deuterium atoms should have been preserved in the direct reduction of the starting allylic alcohol. The alcohol dehydrogenases, which are present in the fermentation medium, catalyse the oxidation of the allylic alcohol to the unsaturated aldehyde, which is easily reduced by ERs and removed from the equilibrium. Then, the saturated aldehyde is further reduced by ADHs to afford the corresponding saturated alcohol. The intermediate aldehydes did not accumulate in the reaction medium and it was not possible to detect them during the reaction course by GC/MS analysis. On the basis of these results, the reaction sequence shown in Fig. 2C can be hypothesized for MCA.

As for CE, the transcripts level of the 10 putative *mcoyes* was monitored both in the presence and absence of MCA at 30 min, 1 h, 2 h and 4 h (Fig. 4B). *Mcoye2* showed the highest gene activation level with about 2,880 fold compared to the control sample, followed by *mcoye1* that displayed 1,860 fold induction (Fig. 4B). An induction of gene expression of about 500 fold was observed for *mcoye4*, *mcoye5* and *mcoye10*. Remarkably, for these 5 genes the highest activation was reached 1 h after the addition of the substrate. For *mcoye3* and *mcoye6* the highest induction levels (138 and 243, respectively) were observed at 2 h. *Mcoye9* displayed moderate gene activation (about 14 fold) only at 4 h, *mcoye7* and *mcoye8* did not show activation, as it was observed with CE (data not shown).

In this case too, a relation between *mcoyes* gene expression activation and MCA biotransformation was observed (Fig. 3B). The strongest activation of *mcoye1* in presence of MCA was reached at 1 h, well before the beginning of the C=C reduction, represented by the formation of the saturated alcohol.

#### Methyl cinnamate (MCI)

The MCI substrate was completely reduced by *M. circinelloides* within 66 h; both the C=C double bond and the ester group were reduced producing cinnamyl alcohol and phenylpropanol. The exact reaction profile is unknown; however, the one reported in Fig. 2D can be hypothesized on the basis of what has been observed for MCA, starting from the enzymatic hydrolysis of the ester moiety followed by the biocatalysed reduction of the COOH group to primary alcohol. As shown in Fig. 3C, MCI decreased slightly but constantly until 66 h, when all the substrate was transformed. The detected amount of cinnamyl alcohol was never more than 20%; also the level of phenylpropanol remained low (<20%) until 9 h, after which the concentration reached 100% within 66 h.

The transcripts level of the 10 putative *mcoyes* was analyzed in presence and absence of MCI at 1, 2, 3 and 6 h (Fig. 4C). *Mcoye1* was the most induced gene (about 300 fold). An activation of about 60 fold was observed for *mcoye2*, *mcoye5*, and *mcoye10*, while *mcoye3*, *mcoye4*, *mcoye6*, and *mcoye9* showed 20 fold induction. *Mcoye1* and *mcoye6* displayed the fastest activation, with the maximum within the first 2 h; after that time point, their transcripts level rapidly decreased. *Mcoye10* showed a peak between 2 and 3 h (Fig. 4C). Also in this case, neither *mcoye7* nor *mcoye8* were activated upon substrate exposure (data not shown).

A relation between OYE activation and the biotransformation of MCI was observed (Fig. 3C). The transcription of *mcoye1* started early, when the substrate was still the only detectable compound in the reaction mixture.

## Discussion

Hydrogenation of C=C double bonds is an important reaction in several manufacturing processes for the production of bulk and fine chemicals; researchers and industries are moving towards more sustainable approaches as biocatalysis and in recent years, several research groups have focused on the identification of OYEs homologues to be exploited in different processes^1^. In the last few years the attention was given to OYEs from filamentous fungi; nevertheless only few studies report their occurrence and their physiological role in this group of organisms^9,13^.

An *in silico* approach allowed to identify in the genome of the zygomycete fungus *M. circinelloides* 10 gene sequences that shared similarity and conserved domains with known OYEs. The presence of multiple OYE genes appears to be a common feature not only among Ascomycetes and Basiomycetes^7,12^ but also in Zygomycetes: indeed with a similar approach we found from 4 to 10 putative OYE sequences within some of the completely sequenced genomes (Suppl. Table 2).

A phylogenetic analysis grouped the McOYEs in two classes: nine proteins (McOYE1–9) were placed in Class I, including most of the OYE1-like proteins^5,9,12^, while McOYE10 clustered with Class II. Genome sequence data allowed to hypothesize that a number of Class I McOYEs are located within the same chromosome; this information may suggest duplication events for some of these genes, as suggested by Corrochano *et al*.^14^. Class II gathers OYEs originally identified from different thermophilic bacteria;^5,9^ however, the recent work by Nizam *et al*.^9,12^ demonstrated that a number of sequences, although not yet characterized, from filamentous fungi (Ascomycota and Basidiomycota) also clusters within Class II. To the best of our knowledge, this is the first report of an OYE homologue from a Zygomycota belonging to this Class.

The fungal enzymatic activity was analyzed in the presence of three different substrates while previous works considered only one substrate or a series of compounds belonging to the same chemical class^15^. *M. circinelloides* showed a strong enzymatic activity being able to completely reduce the C=C double bond of the three substrates. CE was converted very fast (3.5 h), followed by MCA (20 h) and MCI (66 h), suggesting an increasing recalcitrance of the molecules. These results are in line with those obtained by Gatti *et al*.^2^, who demonstrated that the carbonyl moiety acts as a strong activator, while the ester group is a weak EWG. Being able to convert compounds with different EWGs, *M. circinelloides* was very versatile; during the biotransformation the EWG influenced only the timing of the reaction; the ester group of MCI was the weakest EWG as the reaction was accomplished in 66 h.

The reduction of α,β-unsaturated ketones has been extensively studied using either the whole microorganism or the purified enzymes^5,15,16^. Generally CE is a well reduced compound; in fact *M. circinelloides* completely reduced the C=C double bond (100%) in only 3.5 h. Comparable yields were achieved with other filamentous fungi: a previous study, which examined 28 filamentous fungi for the reduction of three different conventional compounds, showed that CE was the easiest to reduce for almost all the fungi (96.4%); in particular, 19 fungi completely reduced this molecule^13^. Stueckler *et al*.^7^ reported that purified OYE1 (*S. pastorianus*) reduced 92% of CE and purified YqjM (OYE from *Bacillus subtilis*) reduced 85% of CE.

The reduction of α-substituted cinnamaldehydes is very important at industrial level^2^. Aldehyde is considered a good EWG and MCA was completely reduced within 20 h; Fardelone *et al*.^17^ obtained comparable yields using a commercial strain of *S. cerevisiae* in the biotransformation of cinnamaldehyde derivatives. Other authors reported that MCA is not always an easily reduced compound. For instance, Goretti *et al*.^18^ analyzed different non conventional yeasts in the reduction of MCA and found that only *Kazachstania spenceroum* was able to convert this substrate with a yield of 60%. Romagnolo *et al*.^13^ reported that, among 19 fungi tested, only two, belonging to the *Mucor* genus were able to completely convert the C=C double bond of this substrate.

The bioreduction of MCI and its derivatives is not frequently reported in the literature, suggesting a possible recalcitrance of this molecule to OYE-mediated biotransformation. A biotransformation study performed on 7 bacterial, yeast and plant OYEs homologues showed a conversion rate of MCI < 1%^19^. Therefore, the ability of *M. circinelloides* to completely reduce MCI is remarkable, since unsaturated esters with no other EWG are rarely converted by OYEs.

BlastP analysis, using OYE1 of *S. pastorianus* as query, allowed the identification of 10 putative genes coding for OYEs, confirmed by PCR amplification and sequencing. The high versatility found in the reduction of different compounds by *M. circinelloides* may depend on its enzymatic pattern and on the possibility to activate distinct genes specifically in the presence of different molecules or in defined environmental conditions. In a recent paper, Nizam *et al*.^9^ performed a genome-wide analysis on available genomes of filamentous fungi: 60 species were investigated leading to the identification of 424 OYE homologues. Surprisingly, some species were shown to possess up to 22 OYEs homologues in their genome, while, in other microorganisms the number of OYEs homologues was more exiguous: only two homologues are present in *S. cerevisiae*, while there are four in *Shewanella oneidensis* ^20,21^.

Gene activation upon exposure to CE and MCA was extremely high (i.e. up to 2,900 fold for *mcoye2* in presence of MCA) and occurred soon after substrate addition. Nizam *et al*.^9,12^ monitored the expression profile of 6 OYEs homologues from the *Ascochyta rabiei* in two different conditions reporting an increase of 80 fold in transcript levels during plant infection and a weaker activation during oxidative stress.

Among the 10 genes identified in *M. circinelloides*, *mcoye1* and *mcoye2* showed the highest degree of gene activation (70–2,900 fold), followed by *mcoye4*, *mcoye5* and *mcoye10* (20–800 fold). *Mcoye3*, *mcoye6* and *mcoye9* were poorly activated, while transcripts of *mcoye7* and *mcoye8* were never activated in each condition. On the basis of these results it seems reasonable to conclude that 8 out of 10 putative OYEs homologues are rapidly activated in response to the substrates addition.

A relation between the biotransformation of each substrate and the expression profile of the eight putative OYEs homologues has been observed. Generally, the transcript levels reached the maximum peak before the beginning of the C=C double bond reduction. For example, during CE analysis, the maximum peak of expression of *mcoye2* was reached after 1 h when 20% of substrate was reduced.

The biological role of these enzymes as well as their cell localization is still an open question. By *in silico* analysis Nizam *et al*.^9^, found that the majority of the OYE homologues were allegedly located in the cytoplasm and in the cytoskeleton, although some of them were associated to other cell compartments such as nucleus, peroxisomes, plasma membrane. Only three OYE seemed to be extracellular. A preliminary experiment carried out on *M. circinelloides* during the biotransformation of CE, showed that ene reductase activity was detected only in presence of cell debris indicating that these enzymes may be intracellular (data not shown); further and deeper experiments are needed to confirm this hypothesis.

Studies are in progress to analyze the secondary and tertiary structure of these enzymes by *in silico* approaches^22^. In order to purify and catalytically characterize McOYEs, efforts will concentrate on the production of the homologues of *M. circinelloides* by heterologous expression systems.

## Materials and Methods

### Fungal strain

*Mucor circinelloides* 277.49 was obtained from CBS (CBS-KNAW fungal biodiversity centre) and was selected due to its capability of reducing C=C double bonds^13^. The strain is preserved as MUT 44 at the *Mycotheca Universitatis Taurinensis* (MUT), Department of Life Sciences and Systems Biology, University of Turin.

### Chemicals

CE, MCA and MCI were purchased from Sigma-Aldrich. Stock solutions of 500 mM of each substrate were prepared in DMSO (Sigma-Aldrich).

(*E*)-2-methyl-3-phenylprop-2-en-1,1-*d*_*2*_-1-ol (dideuterated α-methylcinnamyl alcohol) was prepared by reduction of ethyl (*E*)-2-methyl-3-phenylacrylate (0.50 g, 2.6 mmol) with DIBAL-D (7.9 mmol, 0.7 M in toluene) in THF. After the usual work-up, the dideuterated compound was obtained (0.41 g, 2.3 mmol, 89%). ^1^H NMR (CDCl_3_, 400 MHz): *δ* = 7.39–7.19 (5 H, m, aromatic hydrogens), 6.53 (1 H, q, *Ј* = 1.5 Hz, CH = C), 1.91 (3 H, d, *Ј* = 1.5 Hz, CH3); GC-MS (EI) t_R_ = 14.1 min: m/z (%) = 150 (M^+^, 92), 107 (68), 91 (100). 2-Methyl-3-phenylpropan-1-d-1-ol (monodeuterated α-methyldihydrocinnamyl alcohol) was isolated from the reaction medium and characterized by NMR and GC/MS analysis: ^1^H NMR (CDCl_3_, 400 MHz): *δ* = 7.37–7.13 (5 H, m, aromatic hydrogens), 3.45 (1 H, m, *CH*DOH), 2.75 (1 H, dd *J* = 13.5 and 6.4 Hz, *CH*HPh), 2.43 (1 H, dd *J* = 13.5 and 8.0 Hz, *CH*HPh), 1.97 (1 H, m, *CH*CH_3_), 0.92 (3 H, s, *CH*_*3*_); GC-MS (EI) t_R_ = 12.6 min: m/z (%) = 151 (M^+^, 10), 133 (23), 118 (27), 91 (100).

### Genome mining and phylogenetic analyses

BlastP analysis was performed on the complete genome of *M. circinelloides* strain 277.49 (Joint Genome Institute, JGI: http://jgi.doe.gov) using the sequence of OYE1 of *Saccharomyces pastorianus* (UniProtKB accession no. Q02899) as query. Primer pairs for qRT-PCR assays were designed by using Primer 3 (http://primer3.ut.ee/) (Supp. Table 1). Total genomic DNA was extracted from the mycelium grown in MEA liquid medium (20 g/l glucose, 20 g/l malt extract, 2 g/l peptone) for 24 h using the CTAB method^23^. Oligonucleotides were tested by conventional PCR on genomic DNA. The PCR mixture included distilled water, PCR buffer (10 X), 1 mM deoxynucleotide triphosphates (dNTPs), 10 mM of each primer, 0.5 U of DNA polymerase (Taq DNA polymerase, Qiagen) and 100 ng of genomic DNA in a total volume of 20 μl. Amplifications were performed using a T100 Thermal Cycler (BIORAD). For the validation of *mcoye1* F-R, *mcoye2* F-R, *mcoye3* F-R, *mcoye5* F-R, *mcoye6* F-R, *mcoye9* F-R e *mcoye10* F-R, the amplification protocol was as follows: 95 °C (5 min), 34 cycles of 95 °C (40 sec), 60 °C (50 sec) and 72 °C (50 sec), 72 °C (8 min). For the detection of *mcoye4* F-R, *mcoye7* F-R e *mcoye8* F-R the amplification protocol was as follows: 95 °C (5 min), 34 cycles 95 °C (40 sec), 56 °C (50 sec) and 72 °C (50 sec), 72 °C (8 min). PCR products were loaded on a 1.5% agarose electrophoresis gel stained with ethidium bromide; the molecular weight marker used was the GelPilot 1 kb Plus Ladder (cat. no. 239095, Qiagen). Products were purified and sequenced at Macrogen (The Netherlands). Newly generated sequences were analyzed using Sequencher 5.4 (Gene Code Corporation).

To perform the phylogenetic analyses, over 400 OYEs aminoacidic sequences of fungi were aligned with MUSCLE (http://www.ebi.ac.uk/Tools/msa/muscle/) using default conditions for gap openings and gap extension penalties and trimmed by TrimAl (v 1.2) (http://trimal.cgenomics.org) with the AUTOMATED 1 setting. The analysis was performed using two approaches. First, a phylogenetic tree was derived by Bayesian Inference (BI) implemented in MrBayes (v 3.2.2) (http://mrbayes.sourceforge.net) under a mixed amino acid substitution model. The alignment was run over 10 million generations with two independent runs each containing four Markov Chains Monte Carlo (MCMC) and sampling frequency of every 300 iterations. The first 2,500 trees were discarded as “burn-in” (25%). Using the Sumt function of MrBayes a consensus tree was generated and posterior probabilities were estimated. In a second approach, Maximum Likelihood (ML) was performed using RAxML GUI (v 1.5 b)^24^ with WAG + I + G model. Statistical reliability was determined by Bootstrap analysis. All the phylogenetic trees were visualized using FigTree (v 1.4) (http://tree.bio.ed.ac.uk/software/figtree).

### Biotransformation by whole cell system

A conidia suspension of *M. circinelloides* was made from pre-growth mycelium in MEA solid medium (same composition of MEA liquid with the addition of 20 g/l of agar). 10^6^ conidia were inoculated in 100 ml flasks containing 40 ml of MEA liquid medium. Flasks were incubated at 25 °C in agitation. After 2 days, substrates were added (5 mM final concentration), each cultural line was run in triplicate. In addition, biotic controls (in absence of substrates) were set up.

According to previous results (unpublished data), the conversion of CE, MCA and MCI was followed for 24 h, 48 h and 7 d, respectively. Every 2 h, 1 ml of broth and 100 mg of biomass were collected to perform chemical analysis and RNA extraction, respectively. The mycelium was frozen in liquid nitrogen and stored at −80 °C until the analysis.

At any collection time point, pH and glucose content were measured. The concentration of reducing sugars was obtained following the reaction with 3,5-dinitrosalycilic acid assay (DNS)^25^, using a modified protocol as described by Spina *et al*.^26^. At each time point and at the end of the experiment, fungal biomasses were separated from the culture medium by filtration and dried in oven at 60 °C for 24 h to calculate the dry weight.

### Chemical analyses

Samples taken at the different time points were extracted by two-phase separation using 0.4 ml of methyl *t*-butyl ether (MTBE) as solvent; the organic phase was dried over anhydrous Na_2_SO_4_ and analyzed by GC/MS.

GC/MS analyses were performed on an Agilent HP 6890 gas chromatograph equipped with a 5973 mass detector and an HP-5-MS column (30 m × 0.25 mm × 0.25 μm, Agilent), employing the following temperature program: 60 °C (1 min)/6 °C min^−1^/150 °C (1 min)/12 °C min^−1^/280 °C (5 min). The end products of the biotransformations were identified by GC/MS analysis, using authentic commercial samples as reference compounds: (i) cyclohexenone t_R_ = 5.40 min *m/z* 96 (M^+^, 33), 81 (19), 68 (100); cyclohexanone t_R_ = 4.65 min *m/z* 98 (M^+^, 47), 83 (13), 55 (100); cyclohexanol t_R_ = 4.45 min *m/z* 100 (M^+^, 2), 82 (35), 57 (100), (ii) α-methylcinnamaldehyde t_R_ = 14.7 min *m/z* 146 (M^+^, 64), 145 (100), 117 (79), 91 (43); α-methylcinnamyl alcohol t_R_ = 15.5 min *m/z* 148 (M^+^, 50), 115 (63), 91 (100); α-methyldihydrocinnamyl alcohol t_R_ = 13.7 min *m/z* 150 (M^+^, 12), 117 (62), 91 (100); (iii) methyl cinnamate t_R_ = 16.03 min *m/z* 162 (M^+^, 58), 131 (100), 103 (72); cinnamyl alcohol t_R_ = 12.80 min *m/z* 134 (M^+^, 53), 115 (65), 92 (100); phenylpropanol t_R_ = 12.36 min *m/z* 136 (M^+^, 21), 117 (100), 91 (84).

### RNA extraction, first strand cDNA synthesis and quantitative Real-Time PCR experiments

The extraction of RNA was performed from about 100 mg of fungal biomass using the RNeasy Plant Mini Kit (Qiagen). Quantity and quality of RNA samples were checked spectrophotometrically (Tecan Infinite 200, i-control software). After DNase treatment (TURBO DNA-free, Ambion), RNA quality has been tested again and for all the samples, the ratios of absorbance 260/280 were between 1.8 and 2.2. Subsequently they were processed to obtain cDNA with the use of the Super-Script II Reverse Transcriptase (Invitrogen), following instructions.

qRT-PCR were performed with an iCycler iQTM Real-Time PCR Detection System (BIORAD); reactions were carried out in a final volume of 15 μl by using iTaq Universal SYBR GREEN Supermix (BIORAD), specific primers (3 μM; Table 1) and cDNA. For the detection of *mcoye1*, *mcoye2*, *mcoye3*, *mcoye5*, *mcoye6*, *mcoye9* and *mcoye10*, the amplification protocol was as follows: 95 °C (1.5 min), 40 cycles of 95 °C (15 sec), 60 °C (30 sec) and 72 °C (50 sec), 72 °C (8 min). For the detection of *mcoye4*, *mcoye7* and *mcoye8* the amplification protocol was as follows: 95 °C (1.5 min), 40 cycles 95 °C (15 sec), 56 °C (30 sec) and 72 °C (50 sec), 72 °C (8 min). The *M. circinelloides* β-actin encoding gene was used as internal control^27^. The relative expression was calculated using the 2^−ΔΔCt^ method^28^. One-way ANOVA and Tukey’s tests (p < 0.05) were performed to assess the statistical significance of the gene expression data (IBM SPSS Statistics for Macintosh, Version 22.0).

### Availability of materials and data

Authors confirm that all relevant data are included in the article and its supplementary information file.

## Electronic supplementary material

Supplementary information
