# Supplementary material for: Old Yellow Enzyme homologues in Mucor circinelloides: expression profile and biotransformation
Source: Sci Rep. 2017 Sep 21;7:12093. doi: 10.1038/s41598-017-12545-7 (PMC5608841; doi:10.1038/s41598-017-12545-7)
Supplement: Supplementary file 1 — Supplementary information [file 41598_2017_12545_MOESM1_ESM.pdf]

Old Yellow Enzyme homologues in *Mucor circinelloides*: expression profile and biotransformation.

Alice Romagnolo[a], Federica Spina[a], Anna Poli[a], Sara Risso[a], Bianca Serito[a], Michele Crotti[b], Daniela Monti[c], Elisabetta Brenna[b], Luisa Lanfranco[a] and Giovanna Cristina Varese[a]*

Supp. Fig. 1. a) conserved domains for 9 *mcoyes*. b) Conserved domains for *mcoye10*.

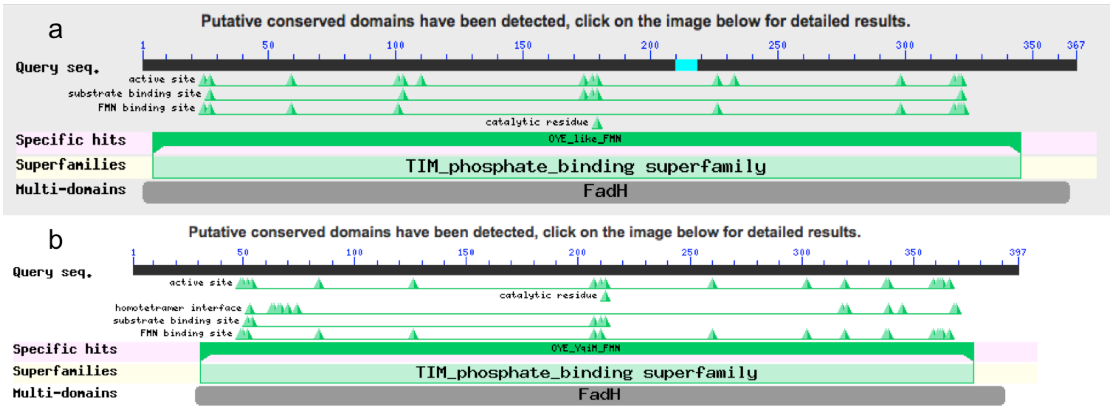

Supp. Fig. 2. PCR products obtained during specific primer validation; b: negative control (PCR mixture with no DNA templates). Molecular weight marker: GelPilot 1 kb Plus Ladder. Image acquired with Gel Doc XR, Biorad and processed with Quantity One software, Biorad.

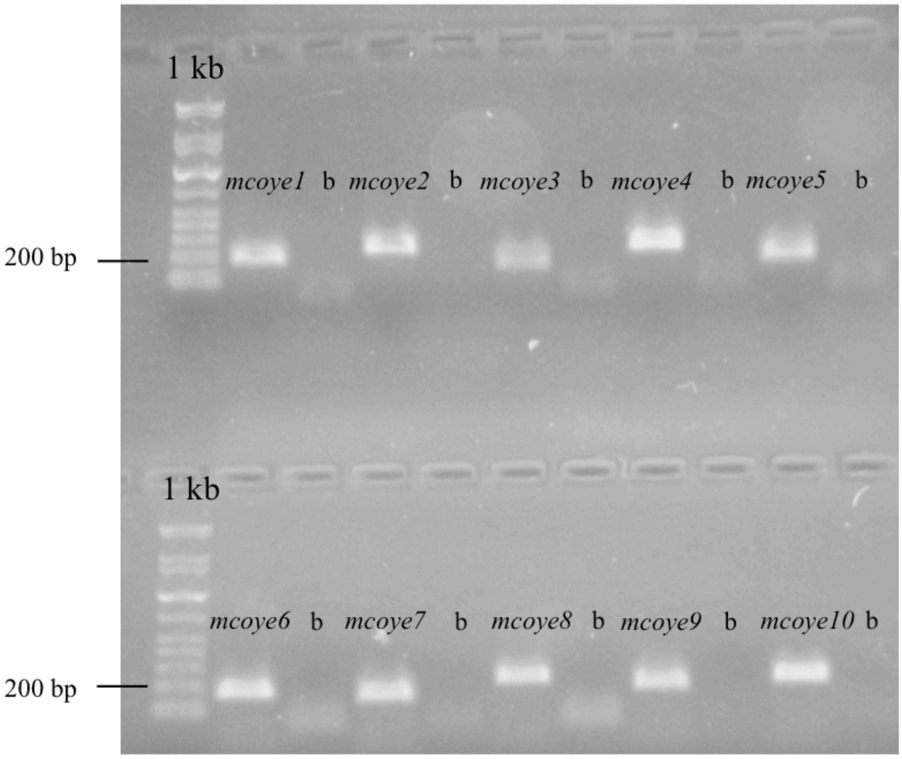

Supp. Fig. 3. qPCR report of three genes (*mcoye9*, *mcoye6* and *mcoye10*). The first three curves represent HK gene. All the genes represented in the graph are expressed after the HK gene.

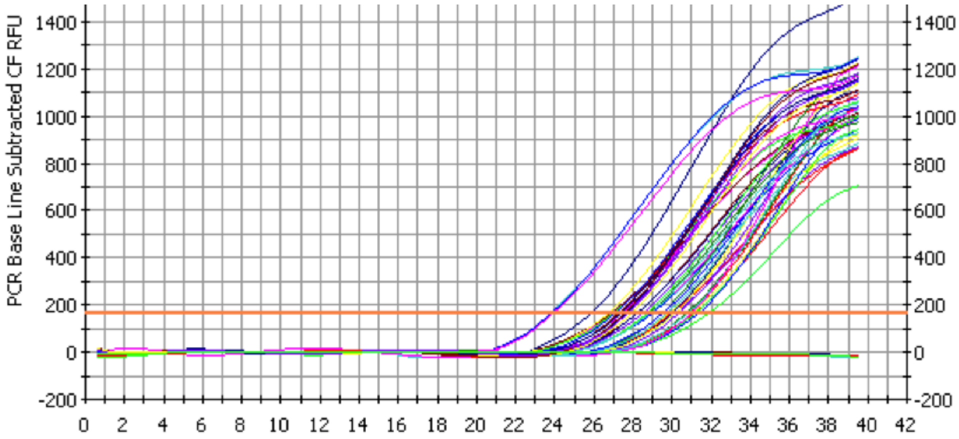

Supp. Table 1. List of specific primers for qRT-PCR of *M. circinelloides*.

Primers	Sequences (5'-3')	Amplicon length	Annealing temperatures (°C)
mcoye1 F	TGCCAATGGTTATCTGGTCG	177	60
mcoye1 R	CTGGAAGCCGTTACCTGGA		
mcoye2 F	TCGTGCCAACTTCTTCTTGC	223	60
mcoye2 R	GGTTCAATTCCCAGCCGTTG		
mcoye3 F	GGCTACTTGACCTCAGAGCT	175	60
mcoye3 R	GAGTCAATGAAAGGCCACCA		
mcoye4 F	AGCTACCTCGTGCTCTTACC	185	56
mcoye4 R	GGATGATCCGCCATAAATATCG		
mcoye5 F	CTCAGGCCCTTCAAGACAGA	157	60
mcoye5 R	GTATCCACCGGCAGAGATGA		
mcoye6 F	GGCGCTTTCATCTACTTGCA	206	60
mcoye6 R	CGCATTTAAGGCAGCTTGCA		
mcoye7 F	TCCCTCCATCATTATCGCAAT	231	56
mcoye7 R	AATCAAGATACCCCACATCG		
mcoye8 F	CTTCTCTCCTAATGGCTCAG	162	56
mcoye8 R	AATGGTGTTCACCTTGCCAT		
mcoye9 F	GTGCAAGATTTTCGCCAAGC	225	60
mcoye9 R	CTCGGATACCTGTTCGCTCT		
mcoye10 F	AATCGGCGTGGATGTCATTG	189	60
mcoye10 R	ATCAGCCTCGTCCTTTTCCA		

Supp. Table 2. Putative OYE homologues of the fungi belonging to the Mucormycotina with sequence ID (JGI database) and identity percentage with *S. pastorianus* OYE1.

Species	Sequence ID	ID % with OYE1
Backusella circina FSU 941 v1.0	239566	52.50
	321291	52.30
	288216	53.60
	326062	53.40
	258711	53.40
	301550	49.30
	320605	53.10
	283228	49.60
	282556	50.00
	270152	51.90
Hesseltinella vesiculosa NRRL3301 v2.0	1409933	49.80
	1344648	48.70
	1389673	52.50
	1407959	46.60
	1276110	51.10
Lichtheimia hyalospora v1.0	204294	53.00
	121608	53.30
	230638	49.20
	167093	37.70
Phycomyces blakesleeanus NRRL1555 v2.0	113057	52.60
	24356	53.10
	112394	52.20
	146092	54.00
	155499	50.60
	133910	51.90
	181382	51.50
	168981	50.40
	168987	50.40
	168986	50.00
Rhizopus microsporus ATCC11559 v1.0	229508	60.20
	224787	51.90
	193785	51.90
	224132	48.70
	226116	60.00
	228303	39.00
	171195	50.70
Rhizopus microsporus var. *chinensis* CCTCC M201021	10823	55.10
	7780	60.20
	5247	59.80
	11257	53.70
	9553	53.10
	15357	51.90
	9473	52.50
	2454	49.10

	579	48.70
	13117	39.00
Rhizopus microsporus var. *microsporus* ATCC52813 v1.0	236210	59.50
	277442	54.00
	196704	52.30
	263891	52.70
	233319	51.30
	210812	51.30
	220486	48.70
	231713	48.00
	198995	39.10
	294582	50.70
Rhizopus microsporus var. *microsporus* ATCC52814 v1.0	1405	59.50
	226692	54.00
	234533	52.70
	221217	51.30
	20582	48.70
	338611	51.90
	323344	45.60
	278916	50.70
	209982	57.10
	301704	49.10
Rhizopus oryzae 99-880	11141	53.30
	13469	53.20
	13470	54.10
	1911	52.90
	13455	52.70
	16652	52.40
	16660	52.90
	5596	36.20
	16962	34.90
	16961	34.90
Umbelopsis ramanniana AG v1.0	167996	50.20
	296378	51.30
	238239	52.10
	228649	33.70
	228848	44.20
	263903	44.30

Biomass dry weight, pH and reducing sugars

The dry weight of fungal biomasses did not change significantly throughout the biotransformation of CE and MCA and did not reveal any remarkable difference between the condition with the substrate and the control either. In the condition without MCI, the biomass remained stable during the whole experiment while, by contrast, after 4 d of MCI exposure there was a slight inhibition of fungal growth (- 30 %).

In all cultural conditions, the pH was acidic (CE: 3.8-4.2, MCA: 4.2-4.7, MCI: 4.1-4.8) and did not display any significant variation throughout the experiments.

The consumption of reducing sugars followed a similar trend for the three substrates; the fungus used most of the glucose (80 %) during the pre-growth phase leaving at the end of the assay, less than 5 %. The consumption profiles in presence and absence of the substrates were similar.
